# The effectiveness of an aged care specific leadership and management program on workforce, work environment, and care quality outcomes: design of a cluster randomised controlled trial

**DOI:** 10.1186/1748-5908-8-126

**Published:** 2013-10-25

**Authors:** Yun-Hee Jeon, Judy M Simpson, Lynn Chenoweth, Michelle Cunich, Hal Kendig

**Affiliations:** 1 Sydney Nursing School, The University of Sydney, 88 Mallett Street, Camperdown, NSW 2050, Australia; 2 School of Public Health, The University of Sydney, A27 Edward Ford Building, Camperdown NSW 2006, Australia; 3 Faculty of Health, University of Technology Sydney, Broadway, Ultimo NSW 2007, Australia; 4 Faculty of Medicine, University of New South Wales, Randwick NSW 2031, Australia; 5 NHMRC Clinical Trials Centre, Sydney Medical School, The University of Sydney, The University of Sydney, Camperdown NSW 2006, Australia; 6 Centre for Research in Ageing, Health and Wellbeing, Research School of Population Health, the Australian National University, Bld 62A, ANU, Canberra, ACT 0200, Australia

**Keywords:** Cluster randomised controlled trial, Aged care, Leadership, Management, Work environment, Quality and safety, Workforce retention

## Abstract

**Background:**

A plethora of observational evidence exists concerning the impact of management and leadership on workforce, work environment, and care quality. Yet, no randomised controlled trial has been conducted to test the effectiveness of leadership and management interventions in aged care. An innovative aged care clinical leadership program (Clinical Leadership in Aged Care − CLiAC) was developed to improve managers’ leadership capacities to support the delivery of quality care in Australia. This paper describes the study design of the cluster randomised controlled trial testing the effectiveness of the program.

**Methods:**

Twenty-four residential and community aged care sites were recruited as managers at each site agreed in writing to participate in the study and ensure that leaders allocated to the control arm would not be offered the intervention program. Sites undergoing major managerial or structural changes were excluded. The 24 sites were randomly allocated to receive the CLiAC program (intervention) or usual care (control), stratified by type (residential vs. community, six each for each arm). Treatment allocation was masked to assessors and staff of all participating sites. The objective is to establish the effectiveness of the CLiAC program in improving work environment, workforce retention, as well as care safety and quality, when compared to usual care. The primary outcomes are measures of work environment, care quality and safety, and staff turnover rates. Secondary outcomes include manager leadership capacity, staff absenteeism, intention to leave, stress levels, and job satisfaction. Differences between intervention and control groups will be analysed by researchers blinded to treatment allocation using linear regression of individual results adjusted for stratification and clustering by site (primary analysis), and additionally for baseline values and potential confounders (secondary analysis). Outcomes measured at the site level will be compared by cluster-level analysis. The overall costs and benefits of the program will also be assessed.

**Discussion:**

The outcomes of the trial have the potential to inform actions to enhance leadership and management capabilities of the aged care workforce, address pressing issues about workforce shortages, and increase the quality of aged care services.

**Trial registration:**

Australian New Zealand Clinical Trials Registry (ACTRN12611001070921)

## Background

Most older people in their sixties and seventies actively contribute to the social and economic fabric of society, and maintain reasonably healthy, independent lifestyles without assistance in their personal and everyday activities [1]. However, extended longevity is accompanied by increasing health issues, in particular chronic diseases and multi-morbidity, and consequently there is a growing need for aged care services, both in residential and community settings [2-5]. The rising expectations of service standards among older people and their families further create greater demands on health and aged care services, managers and staff. Along with the increasing care needs of an ageing population, there is a continued demand for therapeutic nursing care to be provided or supervised by a registered nurse. However, the ability of aged care services to provide high-quality care to this population is often challenged due to a number of aged care workforce issues, including inadequate financial and human resources and skill mix, often accompanied by difficulties in recruiting and retaining qualified nurses with the necessary skills [4,6].

Over time, these issues have led to a substantial change in the aged care workforce profile, where aged care services are now increasingly staffed by care workers (non-licenced workforce) who have limited knowledge or skills in aged or chronic care [7,8]. Changes in the workforce profile have also resulted in a decrease in numbers of managerial staff available to supervise and provide leadership within the sector [9]. Staff shortages, low levels of staff education, low levels of skills in the speciality area, and reduced career prospects for aged care staff compared with their acute and community counterparts, all combine to influence the quality of care services provided in the aged care sector. Such trends are not limited to Australia [10,11].

There is a growing recognition of the need to place a greater emphasis on the quality of individual and organisational leadership capabilities in aged care [12], given their impacts on care quality and safety and staff outcomes [13,14]. Creating a supportive work environment is one of the key strategies to improve staff retention in a sector marginalised by ageist social attitudes [8]. A supportive work environment promotes a strong service mission where staff have ‘adequate supervision, access to professional and emotional support, the establishment of systems that provide feedback to staff (such as regular staff appraisal), and the presence of strong professional leadership’ [8], p.55. National and international evidence point to the significant influence of leadership and management skills on staff turnover, retention and job satisfaction [8,15,16], quality of care, patient outcomes and organisational efficiency [17,18]. The high costs of poor leadership and unhealthy work environments are shown in staff dissatisfaction, absenteeism, and high turnover [19]. Poor leadership has been linked to high staff turnover, but it is yet to be established that poor leadership and high staff turnover relate synergistically to reduce care quality and increase service costs, such as the additional costs of treating urinary tract infections and pressure sores in aged care residents [19-21].

The purpose of this manuscript is to describe the study design and methods of the first cluster randomised controlled trial of an aged care specific leadership program (CLINICAL Leadership in Aged Care – CLiAC). The CLiAC program was developed in recognition of the issues outlined above and the need to focus on the quality of clinical, individual and organisational leadership and management capabilities in both residential and community aged care services in Australia. The aim of the study is to determine the effectiveness of a clinical and managerial leadership program in aged care in improving work environment, workforce retention, as well as care safety and quality, which have not been previously researched in the aged care sector.

### The CLiAC intervention

Leadership is a broad term with a diverse range of applications depending on the position and role of leaders/managers. In aged care services, the middle manager’s role involves ensuring that care quality and safety occur through timely and detailed assessment of care recipients’ health, development of treatment plans, and supervision of nursing staff and care workers [22]. These role responsibilities suggest that leadership capabilities optimise the middle manager’s use of positional authority in leading others to achieve high-quality service outcomes. Yet little evidence exists about the best ways to enable middle managers of aged care services to develop the leadership and management skills that are critical to the effective delivery of high-quality care [23].

The CLiAC aims to achieve safe, high-quality person-centred and evidence-based care by assisting middle managers to develop effective team relationships and person/client-centred leadership strategies that enable them to deal with the day-to-day realities of care service. The Aged care Clinical Leadership Qualities Framework (ACLQF), which was developed from a comprehensive narrative synthesis of leadership and management literature [11] and validated as part of a larger action research project (manuscript under review), underpins the CLiAC program. The findings of the review [11] and the Clinical Excellence Commission Clinical Leadership (CECCL) program [22] further provided the guiding principles of the CLiAC and its implementations, which include that: a leadership program must be incorporated as part of approaches to organisational development and with a strong commitment from the organisation; middle managers are the main target group, but training middle managers should be inclusive of other management levels (*i.e*., senior and frontline managers); and the implementation of the leadership program should occur for 10 to 12 months in which the learning is embedded in the participants’ day-to-day activities.

The CLiAC is a structured manager education and support program that starts with four modules covering core leadership topics delivered in eight full-day workshops. Action learning techniques, 360-degree feedback, case scenarios, one-on-one interactions with a program facilitator, and individual practice improvement projects are included in the 12-month CLiAC program, which is facilitated in the participant’s workplace. The program is designed in such a way that it is congruent with, and incorporated into, the governing organisation’s philosophy, policies, leadership and strategic directions on the grounds that the program delivery requires the organisation’s support, and also that the full potential of effective leadership of middle managers can only be realised when those organisational elements align with the individual’s leadership efforts [23,24].

The CLiAC program is delivered by a facilitator employed specifically for the program by the collaborating organisation, to ensure continuity of the program beyond the study lifetime. The research team and the collaborating organisation worked together to develop the job description for the facilitator. Emphasis was on: first, authenticity of the person who understands the aged care setting and has substantial experience and knowledge in leadership and management; and second, the person’s capacity to be ‘a facilitator and mentor’ rather than ‘an educator.’ The facilitator’s role is to support participants throughout the program via individual meetings and/or teleconferences every four to six weeks, convene group discussions during workshops, provide coaching and participate in peer support meetings. Program participants receive a set of learning resources that include templates for team building activities, developing team-based action plans, providing education sessions, and undertaking the clinical care improvement project.

To enable commitment to the intervention, the facilitator is mentored and supported by an expert education consultant who played the key role in developing the ACLQF and the CLiAC. Weekly contact is maintained throughout the intervention. The facilitator keeps a diary of relevant activities (all formal and informal communication and activity details involving the facilitator and the participants of the CLiAC). The information, with personal details removed, is then sent to the research team on a monthly basis.

## Methods

The study employs a cluster randomised controlled trial designed to comply with the CONSORT guidelines [25]. Cluster randomisation is used because, although managers participate in the CLiAC program individually, they work together at a site to ensure effective aged care is delivered, so the aged care site must be the unit of randomisation. The study is set in urban and rural residential and community aged care services within two states in Australia, New South Wales (NSW) and the Australian Capital Territory (ACT), over three years. The participating research partner is one of the largest aged care service providers on the eastern seaboard of Australia, employing over 4,000 staff across NSW and the ACT. The partner organisation recognised the importance of providing a work environment that optimised staff well-being and increased capacity for high quality, safe care. Clinical leadership for middle management was identified as an area of improvement for the organisation. The organisation agreed to collaborate with the research team in researching these concepts. The collaboration is designed to provide the organisation with the opportunity to build staff capacity in these specific research areas through education/training and practice development. Mutual partnership goals include development of leadership strategies and policies that address workforce recruitment and retention in the aged care sector, which are both considered to be key to progressing high-quality care services.

### Hypotheses

The trial consists of two arms: intervention and control. Managers at the intervention sites agreed to receive the CLiAC program. At the control sites, managers agreed to provide usual care services during the study. The primary hypotheses are that, compared to the control sites, managers and staff at the intervention sites will report an enhanced work environment for staff (H_1_); and improved care quality and safety for care recipients (H_2_); and will have reduced staff turnover rates (H_3_). Secondary hypotheses include that, compared to the control sites, the intervention sites will report: reduced staff absenteeism (H_4_), improved staff ‘intention to stay’ and decreased ‘intention to leave’ (H_5_), reduced stress levels in aged care staff (H_6_), increased job satisfaction for aged care staff (H_7_), reduced costs of retaining and recruiting staff (H_8_), and improved managers’ knowledge and skills in leadership and management (H_9_).

### Ethical considerations

Prior to participant recruitment, research ethics approval was granted by the collaborating organisation’s ethics committee (HREC Code: EC00432), which was then ratified by the Human Research Ethics Committee at the University of Sydney (Database No.13405).

### Participants and sampling frame

Participants are both the care staff and the middle managers of the targeted residential and community aged care services. The sample size calculation was based on the primary outcome, participants’ perceived work environment: the subscales of the Work Environment Scale-R (WES-R) [26]. The study has 80% power to detect a difference of 0.49 standard deviations between groups as significant at the 5% level. This assumes that at least 20 clusters (sites) each with a minimum of 30 participants will complete the study, and that the intra-cluster correlation coefficient (ICC) is 0.26 (average estimate from a recent nursing home staff training intervention carried out in England and Wales) [27], giving a design effect of 8.54. The detectable difference is consistent with the WES-R [26] test developer’s recommendation that half a standard deviation represents a meaningful change on the instrument (Moos, personal communication, Dec. 4, 2008). Consequently, the study will be able to detect a meaningful difference between the trial groups, and it is practical and feasible in terms of study design and data collection.

### Recruitment of targeted aged care sites

Primary study recruitment focused on engaging targeted aged care services to take part in the study and agreement to random allocation of sites to one arm of the study. Secondary study recruitment involved: recruitment of managers at intervention sites to take part in the CLiAC program (and complete evaluation surveys) and recruitment of care staff to complete evaluation surveys; and recruitment of managers and care staff at the control sites to complete evaluation surveys.

Recruitment and follow-up of targeted aged care sites occurred between February 2011 and August 2013 and involved the following three steps:

Step 1. The central executive management of the targeted aged care services provided the research team with a list of eligible services and their contact details based on the site selection criteria (Table 1). A letter was sent to all senior and middle managers of the eligible sites informing them about the study and inviting them to participate.

**Table 1:** Eligibility criteria for the recruitment of aged care study sites and participants

**Recruitment focus**	**Inclusion criteria**	**Exclusion criteria**
**Study sites: aged care services**	1. Principal support for the study is granted in writing from the Executive Care Manager or Community Manager at each site;	1. Sites which are currently (or in the near future will be) undergoing major management/structural changes.
	2. The Executive Care Managers or Community Managers at each site agree in writing that participating managers from their sites who have been allocated to the control group will not receive the intervention program during the study.	
**Participants: middle managers & care staff**	1. Currently work in a permanent middle management or in a direct care role for the participating aged care organisation;	1. Staff involved in non-direct care roles (*e.g*., administration, domestic staff, maintenance, chaplain).
	2. Have been employed by the participating organisation for a minimum of six months;	
	3. If employed in community aged care services, they must be involved in the delivery of aged cared packages including extended aged care at home (EACH), EACH-D (dementia), and community aged care packages;	
	4. Provide consent to take part in the study.	

Step 2. The lead study investigator attended a study-briefing meeting with senior managers from each eligible site to provide detailed information about the study and requirements of participating services. It was emphasised during the meeting that recruitment to the study did not guarantee that the service/site would receive the intervention program. However, the participating services were advised that if they were randomly allocated as a control site, they would be offered the CLiAC intervention after the study has been completed.

Step 3. Following the study-briefing meeting, sites were randomly selected from the eligibility list and invited to take part in the study until a total of 12 RACFs and 12 CACSs agreed to participate.

A total of 24 sites were recruited to allow for the possibility of up to four intervention sites being disqualified from the study. Sites were disqualified from the study if: all the managers of an intervention site did not participate in the CLiAC program or withdrew from the study; or a senior manager at an intervention site moved on, and their replacement did not support the intervention (this excludes a case in which a new senior manager did not want to participate in the CLiAC program while supporting the program in principle).

### Randomisation and blinding

After recruitment of the 24 sites to the study and collection of baseline (Time 1) data from all sites, the sites were allocated to either the intervention or control arm by a biostatistician (JMS) who was not involved in recruitment, data collection, or contact with the sites. Sites were stratified by type of aged care (RACF vs. CACS). Within each stratum, restricted randomisation was used to balance the groups by: size of the service (number of clients), span of control (care staff to middle management ratio), and geographical location (rural vs. urban) [28]. Location is balanced exactly, while for size and span of control the means of the two groups are allowed to differ by no more than 10% in relative terms (*i.e*., ratio of means between 0.9 and 1.1). Allocation was thus fully concealed. The sites were notified by email and completed a written agreement to abide by the random allocation. Also, in an attempt to reduce bias associated with staff becoming aware of managers attending the CLiAC program, participating managers signed forms agreeing not to discuss with their work teams and staff any group specific activities or training that have occurred, and agreed to maintain blinding until the end of data collection. The members of the research team responsible for data entry and analysis will remain blind until completion of the main analysis.

### Recruitment: middle managers and care staff

Recruitment of aged care middle managers and care staff to the study was based on the eligibility criteria shown in Table 1. All staff employed at each site at each data collection time were eligible to participate. An invitation letter and a set of questionnaires were sent to all eligible staff of the participating sites from the research team. Return of their questionnaire was considered as consent (implied consent).

### Measures and data collection

The primary study outcomes are: work environment for aged care staff, care quality and safety, and staff turnover rates. Secondary outcomes include aged care managers' knowledge and skills in leadership and management, aged care staff absenteeism, intention to stay or leave, stress levels, job satisfaction, and various costs including direct costs of recruiting and retaining aged care staff, and cost of resources used in care delivery covered by the aged care service provider.

### Aged care managers and care staff surveys

The self-administered outcome evaluation survey for managers and care staff combines both previously published and validated scales to measure the outcomes of interest. Table 2 provides details of the outcome measures included in the survey and how they relate to each of the hypotheses. In addition, the evaluation survey collates manager and staff demographic information including: age, gender, country of birth, year that employment commenced at the present service, current site and current job role, current title, programs involved in, employment status (full/part-time), professional background, experience in aged care/dementia care (years), highest level of education, aged care specific training, the type of dementia/aged care training or education completed, and the type of leadership/management training or education completed. The surveys were administered at baseline (Time 1), and repeated nine months from baseline (Time 2) and nine months after completion of Time 2 (Time 3). At Time 3, an additional two open-ended questions were added to the staff survey asking them to describe their experience of leadership and management at their site over the past six months and whether they have noticed any changes, plus a general comments question.

**Table 2:** Outcome measures

**Instrument**	**Description of the instrument & domains**	**Hypotheses & measures**
Approaches to Dementia Questionnaire (ADQ) [29]	19 items that measure staff attitudes toward dementia care that reflects their understanding of the need to provide person-centred care for people with dementia. Likert scale ranging from 1 to 5 (strongly agree/disagree). Higher scores indicate a greater understanding of the need to provide person-centred care for people with dementia.	H_2_ – Care quality
Person-centred Care Assessment Tool (P-CAT) [30]	13 items that measure the extent to which staff rate their residential aged-care setting to be person-centred and providing best quality care for people with dementia. Responses are on a Likert scale ranging from 1 to 5 (‘disagree completely’ to ‘agree completely’). Higher scores indicate higher person-centred and quality care.	H_2_ – Care quality
Workforce Dynamics Questionnaire (WDQ) [31]	58 items across 11 domains that measure staff satisfaction, staff perceptions of care quality, access to technology and equipment and training and career progression opportunities. Responses are on a Likert scale ranging from 1 to 10 (‘strongly disagree’ to ‘strongly agree’). Higher scores indicate greater overall job satisfaction and greater perceptions of the quality of care provided, access to technology and equipment, training and greater perceived career progression opportunities.	H_5_ Intention to stay and to leave
		H_7_ – Job satisfaction
Work Environment Scale-R (WES-R) [26]	90 items that measure the perception of the respondents workplace environment including: (a) relationships (involvement, co-worker cohesions, supervisor support); (b) goal orientation (autonomy, task orientation, work pressure); and (c) system maintenance and change dimensions (clarity, managerial control, innovation and physical comfort). True or False response (1 = positive, 0 = negative, total ranging 0 to 90). Higher scores indicate a more positive perception of the workplace environment.	H_1_ – Work environment
		H_6_ – Stress levels
		H_7_ – Job satisfaction
Multi-factor Leadership questionnaire (MLQ)- Manager and Staff version [32]	46 items across 10 subscales that measure different types of leaders and differentiates between effective and non-effective leaders on a 5-point Likert scale ranging from (0 = not at all, 1 = once in a while, 2 = sometimes, 3 = fairly often, 4 = frequently, if not always). Individual aggregate scores for each of the leadership styles are calculated and higher scores indicate a greater tendency towards the particular style. An aggregate score for outcomes of leadership is also calculated, whereby higher scores indicate better perceived leadership including greater effectiveness and satisfaction.	H_1_ – Work environment
		H_9_ – Managers’ knowledge and skills in leadership and management

Distribution and collation of the baseline evaluation surveys occurred in two ways. First, a designated manager at each site was sent survey packs (including invitation letter, study information sheet and survey) to distribute to staff. Staff were then able to return completed surveys in a supplied prepaid self-addressed envelope to the research team. For sites with low response rates, a follow-up telephone call or email was sent to the managers and additional survey packs disseminated as required. Managers were requested to encourage staff to complete the survey, but it was made clear to the managers and staff that participation was not compulsory. Secondly, site visits by a member of the research team were also used to disseminate and collect the evaluation surveys during staff meetings or breaks. During the site visits, staff were able to complete the survey during work hours. Any staff members who were not present during the visit were given the survey to complete and return later.

### CLiAC program evaluation (H_9_ – Manager’s knowledge and skills in leadership)

Key questions from the NHS Leadership Centre Evaluation Questionnaire [33] and the CECCL Evaluation [22] were adapted for the evaluation of the CLiAC program concerning the impact and outcomes associated with the program. The final survey consists of a mix of quantitative questions using various Likert scale responses to review changes associated with the program including: managers’ perceptions of the CLiAC, perceived effects on quality of care, changes to practices and procedures, sustainability of change, improvements to and valued aspects of the program. The survey also has a number of open-ended questions that allow respondents to give feedback on how support for participants of the program might be improved and how the program could be improved overall. The survey takes approximately 40 minutes to complete, and was distributed at 3 months, 6 months and 12 months following completion of the program.

### Collation of human resource data (H_3, 4_ & _8_ – Staff turnover, absenteeism and costs)

Human resource data collected included information on staffing levels, resignations, absenteeism and recruitment. The information was supplied in aggregate form for each site (intervention and control) that took part in the study, for the duration of the study, by the collaborating organisation using a standardised template.

### Collation of data on quality indicators (H_2_ – Care quality and safety)

Quality indicator data included information on the number of unplanned hospital admissions, falls with injury, unintentional weight loss greater than two kilograms, new pressure areas, and new urinary tract infections [34]. These indicators were chosen for their appropriateness and relevance to both community and residential care settings and the fact that they were already part of the regular data collection of the collaborating organisation. Table 3 provides details of the clinical indicators. The data was collected monthly throughout the study (for a total period of two years) using a standardised spreadsheet. For each quality variable, a common set of criteria was detailed and provided to each site along with the spreadsheet. Senior management at all study sites collated the quality indicator data on the last day of each month and sent the spreadsheet to the research team.

**Table 3:** Clinical indicators

**Indicators**	**Definition and criteria**
**Fall with injury**	A fall is an event that results in a person coming to rest inadvertently on the ground or floor or other lower level. If a client is found on the floor or ground, it should be assumed that they have fallen (unless they are cognitively unimpaired and indicate that they put themselves there on purpose). Signs of injury may include fracture, bruises, skin tears, sprains, lacerations, reddened areas or aggravation of pre-existing complaints such as back pain.
**New urinary track infection**	If the resident does not have an indwelling catheter, they must have at least three of the following signs or symptoms:
	1. Fever (greater than 38°C) or chills;
	2. New or increased burning pain on urination, frequency or urgency;
	3. New flank or suprapubic pain or tenderness;
	4. Change in the character of urine;
	5. Worsening of mental or functional status;
	6. New or increased incontinence.
	If the resident has an indwelling catheter, they must have at least two of the following signs or symptoms:
	1. Fever (greater than 38°C) or chills;
	2. New flank or suprapubic pain or tenderness;
	3. Change in the character of urine;
	4. Worsening of mental or functional status.
**New pressure areas**	A pressure ulcer is defined as ‘any lesion caused by unrelieved pressure, resulting in damage of the skin and underlying tissue.’ Alternative terms include ‘bed sore’ and ‘decubitus ulcer.’ The record is kept for occurrences of new pressure areas regardless of their staging or severity.
**Unintentional weight loss**	Weight loss is captured for unintentional weight loss of over 2 kg in any given month. A baseline weight measurement on a specified day within the month needs to be taken before commencing data collection, and then the person is reweighed every month from that date to assess unintentional weight loss. Where a client is on a weight loss diet, due to health reasons, they are not included in this data capture, as we are interested in only unplanned/unintentional weight loss.
**Hospital admissions**	The number of unplanned client transfers (not the number of clients) to hospital where the client has been admitted to hospital. This refers to an unexpected admission for an unexpected event. For example, fracture of neck of femur, post fall, chest infection. This excludes admission to hospital for management of a chronic disease or condition or elective surgery.

NB: The rates are calculated as the total number of new events over six months divided by the mean clients over six months per site.

Economic data (information on the resources used) for implementing the CLiAC program were supplied by the collaborating organisation as aggregate data for all intervention sites using a standardised template. Key items included the program facilitator’s time, teaching materials, and travel costs. Other costs associated with managers participating in the CLiAC program (*e.g*., manager’s time away from their usual work unit and travel) will not be included in the main economic analysis, as they have already been absorbed in the organisation’s usual costs for education and training.

### Data analysis plan

Baseline data will be analysed descriptively by allocated group. All data will be analysed on an intention-to-treat basis (*i.e*., participants will be assigned to the site where they were employed at randomisation even if they subsequently moved to another participating site). Analyses will be performed by an analyst who was not involved in data collection and is blinded to group identification, using either SAS (SAS Institute, Cary, NC, USA) or Stata (StataCorp LP, College Station, TX, USA), and supervised by a biostatistician (JMS).

For the primary analysis, differences between intervention and control groups in the primary outcome at Time 3 will be analysed using linear regression models adjusted for stratification by type of aged care (RACF/CACS) and clustering by site. Other outcomes, such as those for H_2_ and H_3_, which are measured at the cluster level, will be compared by cluster-level analysis, for example, using a negative binomial model stratified by type. All outcomes will similarly be compared at Time 2 to determine the short-term effect of the intervention. In secondary analyses, potential confounders, both at the cluster and individual level, will also be adjusted for, including baseline values of the outcome variables. Potential individual-level confounders include age, gender, previous aged care training/education, and length of time participants have worked in aged care.

An economic evaluation of the CLiAC program will be undertaken using the costs and outcomes (consequences) data discussed above. The first task will involve identifying, measuring and valuing the relevant costs and consequences of the intervention and usual practice (control group). The total costs include the cost of the CLiAC program facilitator, expert education consultant, and educational material. Quantities of resource use will be measured and unit costs (prices) will be assigned, including the cost of mentoring the CLiAC facilitator and implementing the program, using current pay rates (see HR data provided by collaborating organisation) and commercial rates (prices). The outcomes to be explored in the evaluation are: (a) the scores on the work environment measure (WES-R), effectiveness of leadership style (MLQ), person-centred care (P-CAT), and workforce dynamics (WDQ) assessed by staff; and (b) staff turnover and absenteeism. All outcomes (consequences) will be measured in natural units.

The second task will involve comparing changes in the total costs and total outcomes for these groups. This will comprise an incremental analysis of the costs and consequences of the CLiAC program; comparing the additional costs generated by the program over usual practice with the additional outcomes generated by the intervention in terms of: (a) scores on WES-R, MLQ, P-CAT and WDQ assessed by staff; and (b) staff turnover and absenteeism using the HR data. The results of the costs and outcomes will be presented separately (*i.e*., in a cost-consequence analysis) and in several cost-effectiveness ratios (such as a cost per unit change in staff turnover, cost per unit change in absenteeism).

### Trial status

We are currently in the process of completing data collection. All manager and staff surveys have been collected. We are finalising collection of the final round of Human Resource and clinical indicator data. It is expected that data cleaning and analysis will begin in the next month and will be finalised by December 2013. Two of the 24 sites were disqualified after Time 2 based on Rule 1. So far, on average, 81% of manager surveys and 41% of staff surveys have been returned.

## Discussion

This paper presents the design of a cluster randomised controlled trial to evaluate the effectiveness of the CLiAC program in relation to the primary outcome of perceived work environment. To our knowledge, this study is the first cluster RCT examining the effectiveness of an aged care specific leadership program on staff and care quality and safety in the context of both residential and community sites. Rigorous experimental research in aged care leadership (with applications of well-designed models of education, training and development) is rare in this area, and entirely absent from the community aged care sector [24]. Research on aged care leadership is relatively new, and initially it has borrowed much of its theory from models of professional development, with an understandable preference for change management. A comprehensive review of empirical studies on healthcare leadership found that the aged care sector was a sector struggling to adapt these models to its more complex and very different cultural and professional environment [35]. The development of sustainable, effective aged care leadership and management needs to be located in collaborative, communicative and flexible approaches, informed by systematic communication protocols and procedures, which are associated with the spectrum of staffing variables across job satisfaction, retention and recruitment [35]. However, health and care leadership transmission discourse remains trapped in frameworks ill-suited to healthcare, resulting in much of the leadership activity going ‘under the radar,’ unsupported and unrewarded [36].

For good leadership to be sustainable, it is essential that organisational policies be linked to, and congruent with, leadership development programs [37] that can provide the necessary resources for structural and psychological empowerment [38]. Further, Ellis *et al*. [39] advocate a shared governance model that disperses leadership and encourages autonomous work teams in recognition of the complexity of conditions in that sector, and recommends that organisational changes need to be made before changes in staffing in order to support a sustainable work force in aged care. Shared governance refers to any model of participative management or ‘shared accountability,’ or a process through which all members of a group, community or organisation are encouraged to share in the process of decision-making. It is based on the principles that the success of an organisation is dependent upon the legitimate involvement of members in the planning and decision-making processes of that organisation [40,41]. While sufficient resources, including increased staffing levels and wages, are at the top of their list for ways of improving aged care staffing sustainability, DeCicco *et al*. [38] recommend shared governance as the best leadership model. Buchanan *et al*. [36] also posit that shared governance allows opportunities for wider leadership transmission and is worthy of further research. The CLiAC program pays heed to these recommendations by embedding shared governance in the participant’s learning of leadership styles and in the program’s delivery.

The past 10 years have seen substantial developmental changes in the policy and delivery of aged care in Australia as the Government tries to address some of the most pressing care quality and workforce issues. Two of the Government’s most recent and significant actions are worth noting. The Productivity Commission report, Caring for Older Australians [5], pointed out a critical need for improving care quality and workforce capacity and ensuring transparency and accountability of the Australian aged care system. The Australian Government’s aged care reform package, Living Longer Living Better [42], is geared to change the very landscape of how aged care services are provided, in particular in community care. Pressure for change in aged care is intense, and leadership discourse suggests a movement towards models that are better suited to this sector’s unique workplace environments. The CLiAC study is timely, and was specifically designed for (acting) middle managers in the aged care sector. Its findings may well be able to contribute to the current debate about the best ways to address the most critical issues impacting on care quality and safety in the aged care sector: leadership capacity and management capabilities of the workforce.

## Abbreviations

ACLQF: Aged care specific clinical leadership quality framework; CACS: Community aged care service; CECCL: Clinical excellence commission clinical leadership program; CLiAC: Clinical leadership in aged care program; MLQ: Multi-factor leadership questionnaire; P-CAT: Person-centred care assessment tool; RACF: Residential aged care facility; WES-R: Work environment scale.

## Competing interests

The authors declare that they have no competing interests.

## Authors’ contributions

Y-HJ conceived the study and developed the initial study design, which was further refined by JMS, HK and LC. Y-HJ, HK, JMS and LC secured funding for the study. JMS and MC provided statistical and economic advice for the study, respectively. Y-HJ developed the initial draft of the manuscript, and all authors contributed to critical revision for important intellectual input. All authors read and approved the final manuscript.
